# Enhancing Pullulan Soft Capsules with a Mixture of Glycerol and Sorbitol Plasticizers: A Multi-Dimensional Study

**DOI:** 10.3390/polym15102247

**Published:** 2023-05-10

**Authors:** Kecheng Zhou, Yucheng Yang, Bingde Zheng, Qiqi Yu, Yayan Huang, Na Zhang, Shriram Mourougane Rama, Xueqin Zhang, Jing Ye, Meitian Xiao

**Affiliations:** 1 College of Chemical Engineering, Huaqiao University, Xiamen 361021, China; 2 Xiamen Engineering and Technological Research Center for Comprehensive Utilization of Marine Biological Resources, Xiamen 361021, China; 3 Department of Chemical Engineering and Chemistry, Eindhoven University of Technology, Het Kranenveld (Bldg 14-Helix), 5600 MB Eindhoven, The Netherlands

**Keywords:** soft capsule, plant polysaccharide, plasticizer mixture, glycerol, sorbitol

## Abstract

The plasticizer is crucial in the plant-based soft capsule. However, meeting the quality requirements of these capsules with a single plasticizer is challenging. To address this issue, this study first investigated the impact of a plasticizer mixture containing sorbitol and glycerol in varying mass ratios and the performance of the pullulan soft film and capsule. The multiscale analysis demonstrates that the plasticizer mixture exhibits superior effectiveness in enhancing the performance of the pullulan film/capsule compared to a single plasticizer. Furthermore, thermogravimetric analysis, Fourier transform infrared spectroscopy, X-ray diffraction, and scanning electron microscopy indicate that the plasticizer mixture enhances the compatibility and thermal stability of the pullulan films without altering their chemical composition. Among the different mass ratios examined, a 15:15 ratio of sorbitol to glycerol (S/G) is identified as the most optimal, leading to superior physicochemical properties and meeting the requirements for brittleness and disintegration time set by the Chinese Pharmacopoeia. This study provides significant insights into the effect of the plasticizer mixture on the performance of pullulan soft capsules and offers a promising application formula for future use.

## 1. Introduction

As one of the traditional dosage forms, a soft capsule is often used to wrap liquid drugs [1]. Compared with other dosage forms, the soft capsule has the characteristics of isolating the unpleasant odors and tastes of drugs and protecting the encapsulated compound against oxygen, water vapor, or light [2]. The soft capsule is commonly manufactured from a film-forming agent, gel agent, plasticizer, and other trace excipients [3]. The film-forming agent is the main component of the soft capsule, which mainly comes from animal gelatin [4], which sometimes is not accepted by religious people and is easily carries animal diseases [5]. The soft capsule without gelatin can effectively avoid these defects, hence the plant-based soft capsules have attracted much attention from scholars [6,7,8,9,10,11,12].

Currently, the film-forming agents in the plant-based capsules include three categories: cellulosic fat (such as hydroxypropyl methylcellulose [8], carboxymethyl cellulose [13], nanocrystalline cellulose [14], etc.), polysaccharides (such as pullulan [15], carrageenan [16], etc.), and starch (such as modified corn starch [11], potato starch, etc.). In these materials, pullulan is a linear polysaccharide produced by the fermentation of aureobasidium pullulans, formed by the polymerization of maltotriose repeating units linked by α-1,4 glycosidic bonds through α-1,6 glycosidic bonds [17]. Pullulan has the advantages of being non-mutagenic, odorless, colorless, and having good solubility [15]. Hence, it is widely used in the food, materials, and pharmaceutical industries [18]. Yuanyuan Ding et al. [9] used pullulan as the primary material to prepare hard plant capsules, and the results revealed that the capsule was of adequate size, tightness, brittleness, and leak. 

However, only pullulan is not satisfied in preparing the capsule [9]. Hence, extra components should be blended with it to meet the quality requirements of the capsule. For example, a plasticizer is essential in the pullulan capsule or other films. Previous research shows that pullulan films without plasticizers are hard and brittle [19,20] due to their poor mechanical properties, which does not benefit the preparation of the capsule. Plasticizer always has a small molecule, can weaken the intermolecular forces between polymer chains, increase the free volume of polymer molecules, and produce films with a uniform texture and good flexibility [21,22,23]. Plasticizers can be hydrophilic, such as polyols, fatty acids, and monosaccharides, or hydrophobic, such as citrate esters. Hydrophilic plasticizers are commonly used in polymer films, especially polyols, which are particularly effective in plasticized hydrophilic polymers [24,25]. Glycerol and sorbitol are typical polyol plasticizers with good water solubility that effectively improve mechanical properties, such as tensile strength and elongation at break. Parameswara Rao vuddanda et al. [26] studied the effect of plasticizers on a pullulan film, and they found that adding glycerol can improve the flexibility of pullulan polysaccharide film. Abdulaal Farhan et al. [27] found that both glycerol and sorbitol can increase the mechanical barrier property of the film, and the film with sorbitol is a more effective oxygen barrier and is more uniform and smoother than those with glycerol. Rim Gheribi et al. [28] also studied the effects of plasticizers, such as sorbitol and glycerol, on the plant polysaccharide film, and the results show that adding glycerol improves the flexibility of the film, and adding sorbitol intensifies the mechanical strength and water vapor barrier properties of the film.

Different plasticizers affect the appearance, as well as physical, morphological, and mechanical properties of biopolymer plastic [29]. Hence, adding one plasticizer to meet the quality requirement of the films or the capsules is hard to achieve. Adopting the plasticizer mixture might satisfy the demand for different polymer products for plasticizers while compensating for the deficiency of a single plasticizer. K.Z. Hazrati et al. [30] added a plasticizer mixture of glycerol and sorbitol in the dioscorea hispida starch film, which improved the tensile strength of pure glycerol film and mitigated the brittleness of pure sorbitol film. The results show that the film has better tensile properties and appropriate thermal stability.

Therefore, based on the advantage of the plasticizer mixture, this work studies the effect of pure glycerol, pure sorbitol, and the compounds sorbitol and glycerol at different mass ratios on the performance of the pullulan soft capsules. Thermogravimetric analysis (TGA), Fourier transform infrared spectroscopy (FTIR), X-ray diffraction (XRD), and scanning electron microscopy (SEM) are used to multiscale analyze the characteristics of this pullulan material. The results will reveal the effect of the plasticizer mixture on the performance of the pullulan soft capsule and determine the most reasonable product formula.

## 2. Experimental

### 2.1. Materials

The material chosen for this study was commercially available food-grade pullulan (Freda Biotechnology Co., Ltd., Jinan, China); Food-grade κ-carrageenan and ι-carrageenan (Luxin Food Co., Ltd., Zhangzhou, China); Pure glycerol and sorbitol (Macklin Biochemical Co., Ltd., Shanghai, China); Pure potassium citrate, sodium chloride, and anhydrous calcium chloride (Sinopharm Chemical Reagent Co., Ltd., Shanghai, China).

### 2.2. Solution Preparation

The plasticizer mixture (30%, *w*/*w* based on weight of water) of sorbitol (S) and glycerol (G) at different mass ratios (S/G = 0:30; 10:20; 15:15; 20:10; 30:0) was dissolved in distilled water and continuously stirred for 10 min under a water bath heating at 85 °C to prepare a film-forming suspension. Then, pullulan (30%, *w*/*w*), κ-carrageenan (4%, *w*/*w*), ι-carrageenan (0.75%, *w*/*w*), and potassium citrate (0.3%, *w*/*w*) were successively added in the solution and constantly stirred at the same temperature for 1.5 h until the powders completely disappeared. Finally, the solution was cooled to 75 °C and kept for 4 h to remove trapped air bubbles.

### 2.3. Casting Film Preparation

The pullulan soft films were prepared according to the casting process. A sufficient volume of the above-prepared solution (10 cm × 10 cm) was poured onto the plate, which was then dried for 24 h at a relative humidity (RH) of 45% and an ambient temperature of 30 °C. The dried-up films were kept in polyethylene bags for subsequent measurements.

### 2.4. Soft Capsule Preparation

**Figure 1:**
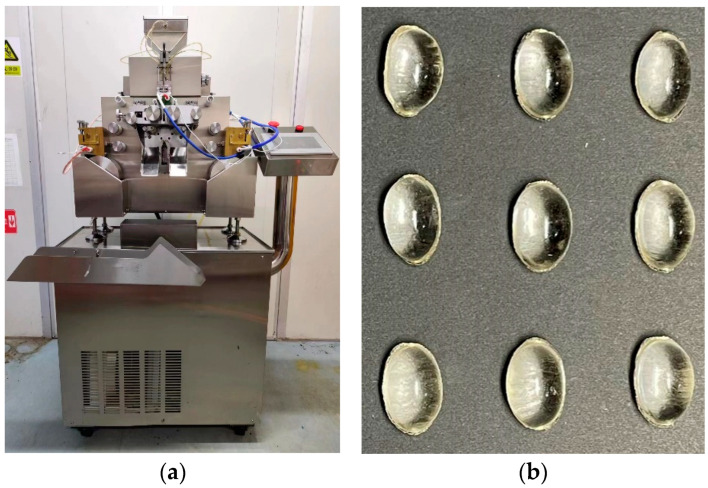
(**a**) soft capsule machine (HSR-60, Beijing, China) and (**b**) soft capsules prepared in this work.

The soft capsule was prepared by transferring the above-prepared solution to a soft capsule machine (HSR-60, Beijing, China), as seen in Figure 1a. The temperature of the gravity-fed spreader box was set at 75 °C to maintain the glue state of the solution. Then, the solution was transferred from the box to the cooling drum, where the rotational speed was set at 0.10–0.21 rad/s, and the temperature was set at 18 °C to ensure that the solution formed intact films. Next, the pullulan soft films were molded using the rotary equipment in the machine. In the meantime, liquid paraffin was added to the pullulan-based soft capsules as the loading material. Finally, the capsules were dried in an oven with an RH of 45% and at a temperature of 30 °C for 24 h.

### 2.5. Characterization

#### 2.5.1. Mechanical Properties of Films

The mechanical properties of the films, including elongation at break (EB, %) and tensile strength (TS, MPa), were evaluated using an electronic tensile testing machine (WZ-03, Guangzhou China), following the procedures specified in the Chinese Pharmacopoeia (11th edition). Specifically, each film sample was cut into a dumbbell shape with a width of 10 mm and a length of 80 mm, ensuring consistency among the samples. The sample was fixed between the device grips with an initial grip separation of 50 mm and cross-head speed of 60 mm/min, with a tensile load cell of 0.5 kN. Each test consisted of three replicate measurements.

TS was calculated by dividing the peak stress (*F*, N) by the cross-sectional area (*A*, m^2^). Percentage EB was calculated by dividing the increase in length at break (*L*) by the initial length (*L*_0_) of the sample and multiplying by 100. It can also described in the following equations:

TS = *F*/*A* (1)

EB = (*L* − *L*_0_)/*L*_0_ × 100 (2)

#### 2.5.2. Water Vapor Permeability of Films

The water vapor permeability (WVP) of the film was determined by adopting a standard cup method [31]. Each film was mounted on the center of the moisture cup (60 mm diameter), containing anhydrous calcium chloride with 5 g. Use sealing wax to seal the groove of the moisture permeable cup to avoid water leakage at the edge and keep it in a 23 °C incubator with 90% relative humidity. The absorption of water through the film was determined by periodic weighing. The change in weight indicated an increase in water. The assembly was weighed at regular intervals of time for 24 h, and WVP was calculated by the following equation:

(3) $$WVP=\frac{\Delta m\cdot d}{A\cdot t\cdot \Delta p}$$

where Δm (g) represents the weight change in the cup before and after the test, d (m) represents the thickness of films, ΔP (Pa) represents the partial pressure difference across the films, and A (m^2^) and t (h), respectively, represent the test area and the test time.

#### 2.5.3. Moisture Sorption Tests of Films

The moisture sorption test of the film was carried out according to the previous method [15]. To keep the relative humidity (RH) of the atmosphere in the glass at 75%, the saturated NaCl solution was placed in a sealed glass at 25 °C. Each film was cut into a square sample (2 cm × 2 cm) and dried at 105 °C until its weight was stable. This weight was recorded as m_0_. Then, the sample was placed on a polypropylene tray in the prepared glass and was intermittently weighed as mt at 2, 4, 8, 24, 48, 72, and 96 h. The moisture sorption rate (MSR) can be calculated according to Equation (4).

Moisture sorption rate = (m − m_0_)/m_0_ × 100% (4)

Each test was done in triplicate.

#### 2.5.4. Thermogravimetric Analysis

The thermal stability and decomposition characteristics of films were measured using a thermogravimetric analyzer (DTG-60, Kyoto, Japan). Each film was cut into small pieces that weighed around 5–10 mg and was put in an alumina crucible. They were heated from 30 °C to 600 °C at a rate of 10 °C/min^−1^ under argon.

#### 2.5.5. Fourier Transforms Infrared Spectroscopy (FTIR)

Fourier transforms infrared spectroscopy (NICOLET iS50, Shanghai, China) was used to obtain the FTIR spectra of films. The ground film (1.0 mg) and KBr powders (100 mg) were placed in an agate mortar to prepare pellets under hydraulic pressure. FTIR spectra of each pellet were performed with a resolution of 4 cm^−1^ and a scanning range of 400–4000 cm^−1^ and 32 scans.

#### 2.5.6. X-ray Diffraction (XRD)

The crystallinity of the film was measured using an X-ray diffractometer (SmartLa, Tokyo, Japan). The scanning range of the diffraction angle 2θ° was 5–40°, with a scanning rate of 5°/min.

#### 2.5.7. Scanning Electron Microscopy (SEM)

Both the surface and the cross-section of the film were observed by scanning electron microscope (SU-5000, Kyoto, Japan). The film was immersed in liquid nitrogen and then fractured into pieces. They were fixed to a sample holder by conductive carbon tape and then sputter-coated with a gold target in a vacuum evaporator. The images were taken at an accelerating voltage of 8 KV.

#### 2.5.8. Analysis of Disintegration of Soft Capsules

The disintegration test for the soft capsules was conducted following the Chinese Pharmacopoeia. Every time, six soft capsules from a batch of samples were placed in the simulated gastric juice in the disintegration tester (ZB-1E, Tianjin, China) at 37 ± 2 °C. The time all six capsules completely disintegrated was recorded as disintegration time.

#### 2.5.9. The Brittleness Test of Soft Capsule

The brittleness test for the soft capsules was carried out according to previous research [10]. The soft capsule sample was placed on the bottom of a stainless tube with a height of 300 mm and a diameter of 33 mm. A stainless-steel weight, weighting 100 g, was dropped to attack the capsule from the top of the tube. The 30 capsules from the same batch of samples repeated the experiment. Meanwhile, the broken soft capsule samples were counted.

## 3. Results and Discussion 

### 3.1. Effect on Mechanical Properties of Pullulan Soft Film

**Figure 2:**
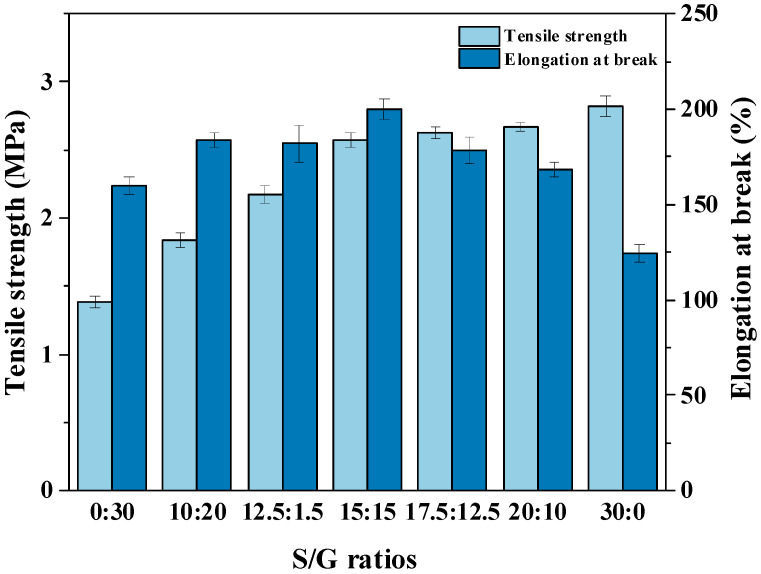
Mechanical properties of pullulan soft films with different S/G ratios.

The reliable mechanical properties of polysaccharide film are crucial to produce high quality soft capsules [9]. Therefore, the effect of different mass ratios between sorbitol and glycerol on the mechanical properties of pullulan soft films are studied, and the results are shown in Figure 2. With the increase in the mass ratio of S/G, the tensile strength of the pullulan soft film improves quickly, but the intensified effect on the tensile strength is no longer obvious after the ratio of S/G passes 15:15. Meanwhile, the growth of the mass ratio of S/G also affects the elongation at break, which increases gradually before the mass fraction of glycerol below half in the plasticizer mixture, and then the elongation at break decreases after that. The reason may be that sorbitol has a longer molecular chain compared to glycerol at the same ratio between hydroxyl and carbon atoms, resulting in forming of a stronger combination between sorbitol and polysaccharide by hydrogen bonds, then increasing the tensile strength and elongation at break of pullulan soft film. However, an excessively high sorbitol content caused high crystallinity and phase separation, reducing the elongation at break of the pullulan soft films [32]. Moreover, Qunyi Tong et al. [19] has shown that the pullulan films without plasticizers exhibit high tensile strength of up to 68 MPA, but only have an elongation at break of 9.6%, suggesting a brittle structure. However, this work has found that the addition of a plasticizer mixture significantly increases the elongation at break of the film, particularly when the mass fraction of glycerol is equal to that of sorbitol. This factor is important because the pullulan film as ribbon may be broken by the rotating drum and the wedge during the encapsulation process. Although the pullulan soft film with only sorbitol as a plasticizer exhibits better tensile strength than the film with the plasticizer mixture, the difference is not significant when the S/G ratio exceeds 15:15.

### 3.2. Effects on Water Vapor Transmittance and Moisture Absorption of Pullulan Soft Film

**Figure 3:**
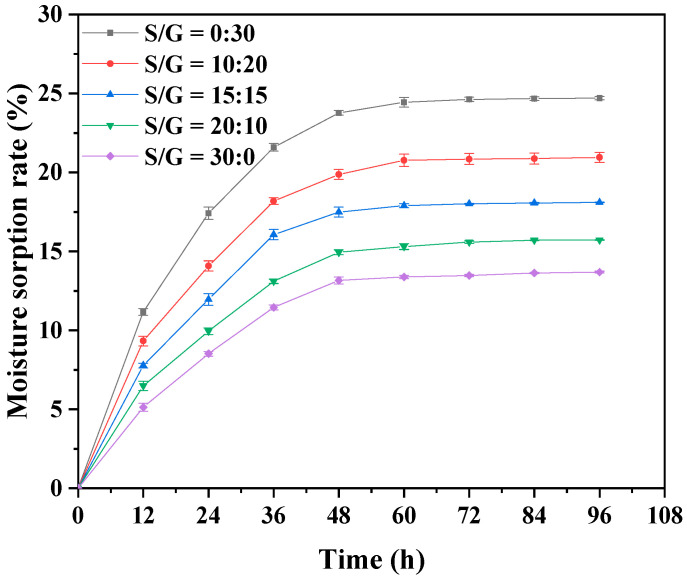
Moisture absorption of pullulan soft films with different S/G ratios.

The performances of the soft capsule in moisture absorption and water vapor transmittance are essential to the storage and application of the capsule. Therefore, the effects of the mass ratio of S/G on moisture absorption and water vapor transmittance of the pullulan soft film are studied, and the results are, respectively, shown in Figure 3 and Table 1. Figure 3 exhibits that the dry basis moisture content of all pullulan soft films in this work gradually increases before the first 60 h, and, after that, they maintain stability, indicating the moisture content in these films reach saturated after 60 h. The saturated moisture content of the pullulan soft film with a single glycerol plasticizer is the most significant compared to other films in this work, which can be explained by the fact that glycerol is more hydrophilic than sorbitol [33]. The small molecular mass of glycerol allows it to more effectively hinder intermolecular and intramolecular hydrogen bonds of the polysaccharide chains, increasing the free volume and thereby enhancing the entry and binding of water molecules. Therefore, the pullulan film with higher mass ratio of glycerol absorbs more water vapor. Moreover, the pullulan soft film with only glycerol has the highest value of WVP in these films. With the increased mass ratio of S/G, the water vapor resistance performance of the film improves. The hygroscopicity of sorbitol is low due to its ability to crystallize at room temperature and high relative humidity [33]. Meanwhile, the molecular interaction between sorbitol and polymer chains increased and became denser, preventing water vapor transmission in the film [32]. Therefore, when considering the formula of pullulan polysaccharide, the procedure with less glycerol content and a higher sorbitol ratio should be selected.

### 3.3. Effects on Thermal Properties of Pullulan Soft Film

**Figure 4:**
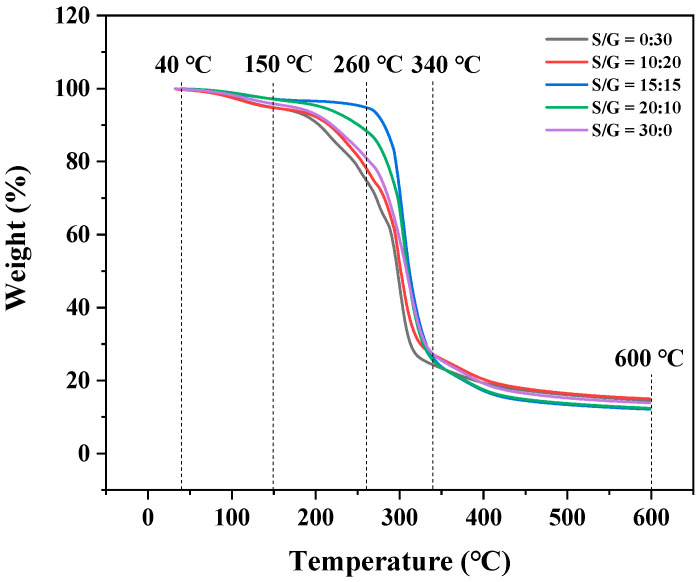
DTG of pullulan soft films with different S/G ratios.

The thermogravimetric analysis (TGA) was used to evaluate the thermal stability of the pullulan soft films at different mass ratios of S/G, and the result is exhibited in Figure 4. The result shows the thermogravimetric curves of these pullulan soft films, which all have a similar thermal degradation behavior. There are three stages for the mass loss of the pullulan soft film during the heating process: the first stage, between 40 °C and 150 °C, is attributed to the evaporation of free water in the pullulan soft films; the second stage, between 150 °C and 260 °C, is attributed to the evaporation of combined water and plasticizer in the pullulan soft films; the third stage, between 260 °C and 600 °C, is attributed to the degradation of the plasticizers and polysaccharides. This trend is similar to that described by previous studies [30,34,35]. The result shows that the flection point of the pullulan soft film at the mass ratio of S/G of 15:15 has the highest temperature, and the flection point of most pullulan soft films with the plasticizer mixture is a higher temperature than that of the film with the single plasticizer. This fact proves that adding a plasticizer mixture can improve thermal stability of the pullulan soft film compared to adding a single plasticizer.

### 3.4. Fourier Transform Infrared Spectroscopy Analysis

**Figure 5:**
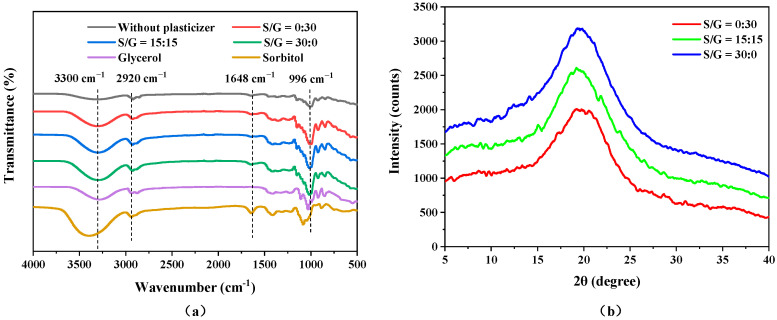
(**a**) FTIR and (**b**) XRD of pullulan soft films with different S/G ratios.

The functional groups of the pullulan soft films, without and with plasticizers, pure sorbitol, and pure glycerol, were investigated using Fourier transform infrared spectroscopy (FTIR), and the results of the FTIR spectra of the six materials are shown in Figure 5a. For the pullulan films, the characteristic peak near 996 cm^−1^ represents the stretching vibration of the glycosidic bond [27], while the absorption peak at 2920 cm^−1^ corresponds to the axial deformation of C-H, which may be caused by the absorption of symmetrical and asymmetrical stretching of -CH_3_ or even groups of -CH_2_ and -CHO [36]. A broad absorption peak near 3300 cm^−1^ is prominent due to the formation of hydrogen bonds by the interaction of O-H groups at the end of the pullulan soft film chains and in the plasticizers [33]. The main functional groups of glycerol are O-H and C-H, which appear at 3300 cm^−1^ and 2920 cm^−1^, respectively. The peak at 1648 cm^−1^ in the sorbitol spectrum is attributed to the stretching of the C=O bond. The FTIR spectra of the pullulan films indicate that the peak strengths at 3300 cm^−1^ and 996 cm^−1^ were enhanced after the addition of plasticizers, indicating that the addition of plasticizers strengthens the hydroxyl bond and glycosidic bond [37]. Moreover, the O-H peak becomes sharper with an increase in the mass ratio of sorbitol, possibly because the hydroxyl connections between pullulan and sorbitol are more sufficient than that with glycerol. Additionally, the results show that the three pullulan films with plasticizers display absorption peaks in similar areas, regardless of the different plasticizer forms and concentrations, demonstrating that all films had identical functional groups because even though the plasticizer types used during the film development are different, all of them are polyols [30].

### 3.5. X-ray Diffraction Analysis

X-ray diffraction (XRD) is used to investigate the crystal structure of the pullulan soft film. The trends in the XRD patterns of pullulan soft films with pure sorbitol, pure glycerol, and their compounds are shown in Figure 5b. Three films have similar trends, with a broad diffraction peak at 2θ = 19.7°. This indicates that these films have a certain degree of ordered structure or fragments, and all have the structural characteristics of a B-type crystal. However, with the addition of sorbitol, the diffraction peak becomes sharper, representing the degree of crystallinity as the film increases. This is because, compared to the connection between sorbitol and glycerol, the connection between glycerol and pullulan is weaker due to the shorter molecular chain of glycerol, resulting in a low degree of crystallinity of the film.

### 3.6. Scanning Electron Microscopy

**Figure 6:**
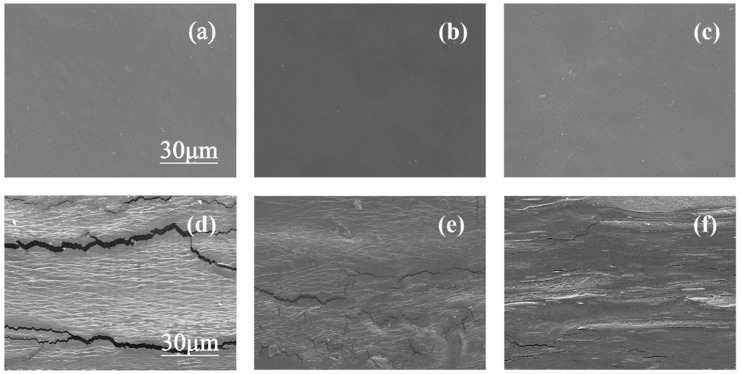
(**a**–**c**) Surface morphology of pullulan soft films with different S/G ratios and (**d**,**e**) cross-sectional morphology of pullulan soft films with different S/G ratios, (**a**,**d**) S/G = 0:30, (**b**,**e**) S/G = 15:15, and (**c**,**f**) S/G = 30:0.

The Scanning electron microscopy (SEM) analysis was carried out to characterize the morphology of the surface and cross-section of the pullulan soft films with pure sorbitol, pure glycerol, and their compounds, and the results are shown in Figure 6. Figure 6a–c show the surface morphology of pullulan soft film, and all the sample surface are uniform and smooth. Figure 6d–f show the cross-section morphology of the pullulan films. Different from the observation of the surface of the film, the cross-sections of the three films have roughness and layering, especially the film with pure glycerol has apparent cracks. This may be due to the weak connection between glycerol and pullulans. Meanwhile, the film with pure sorbitol has a rougher structure than the film compounded with glycerol and sorbitol because the film with pure sorbitol has a higher degree of crystallinity, resulting in phase separation. Therefore, the plasticizer mixture can effectively improve the uniformity of the pullulan soft film.

### 3.7. Effects on Pullulan Soft Capsule Performance

**Figure 7:**
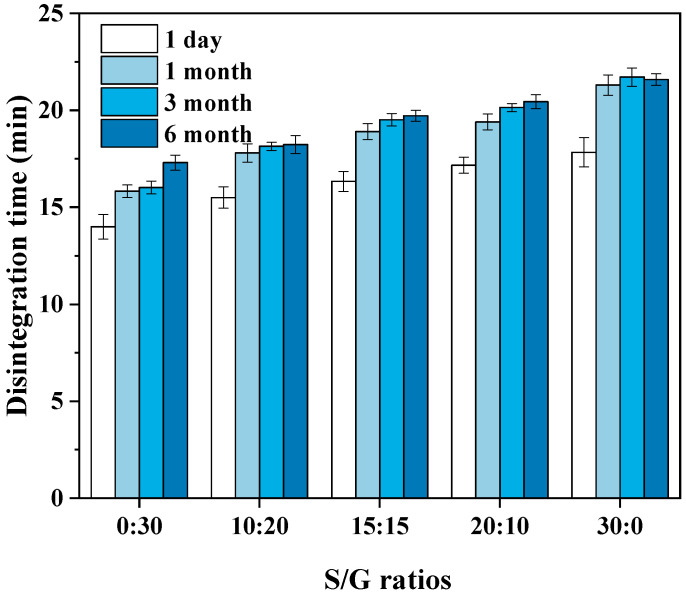
Effect of S/G ratios on disintegration time of soft capsules.

According to the requirements of the Chinese Pharmacopoeia (11th edition), one of the critical indicators of the soft capsule is that the soft capsule should be disintegrated within 60 min. Therefore, the pullulan soft capsules with various mass ratios of S/G and storage time were evaluated based on the disintegration time, and the results are shown in Figure 7. The result shows that the disintegration time of all plant soft capsules in this work is shorter than the limited time. Usually, the shorter disintegration time benefits quick drug release in the human body. The pullulan with a higher mass ratio of S/G has a longer disintegration time, and this can be explained by the above results that the hygroscopicity of sorbitol is lower than the glycerol. Hence, water molecules are difficult to penetrate the capsule, resulting in difficult disintegration. Moreover, with the increase in storage time in a stable environment, the disintegration time of the pullulan soft capsule becomes longer, especially for the film with pure glycerol. The reason for the increase in disintegration time of the capsule during storage may be due to the absorption of water vapor. As the capsule absorbs a certain amount of water vapor during storage, a water balance is achieved inside and outside the capsule. This absorption of water vapor also interact with the -OH groups in the capsule, resulting in a reduction in the number of available -OH groups [10]. Therefore, the disintegration liquid may not be able to penetrate the capsule as quickly without sufficient attraction from -OH groups and blockage of gaps by the absorbed water vapor, ultimately leading to an increase in disintegration time. However, compared with the requested disintegration time of 60 min, the effects of mass ratios of S/G and storage time on the disintegration time of the capsule are obscure.

**Figure 8:**
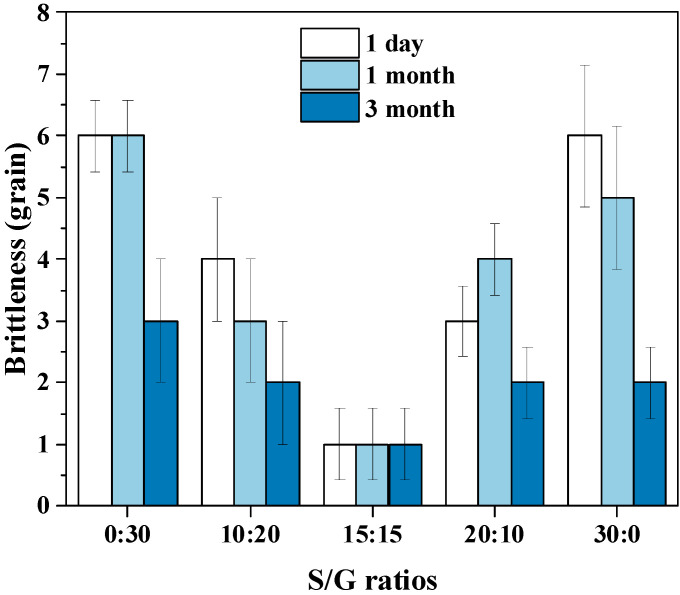
Effect of S/G ratios on the friability of soft capsules.

The friability of the soft capsule is also a critical indicator of a capsule, so the performance of the pullulan soft capsules with different mass ratios of S/G and storage time are shown in Figure 8. The lower friability of the capsule offers better quality, and the product is considered qualified when the friability value is below five, within 30 soft capsules, based on the Chinese Pharmacopoeia. The result shows that the capsule with an S/G mass ratio of 15:15 has the best quality due to the friability of one within 30 soft capsules. Additionally, then, improving or decreasing the mass ratio of S/G increases the friability of the capsule. The result can be provided by the mechanical properties of the pullulan soft film, in which the film with an S/G mass ratio of 15:15 has the best elongation at break and good tensile strength. Moreover, the friability of the capsule decreases with a longer disintegration time because the capsule becomes softer when it absorbs more water vapor from the storage environment. However, the effect of storage time on the soft pullulan capsule with the S/G 15:15 ratio is minimal, which may be due to its excellent mechanical properties.

## 4. Conclusions

The performances of the pullulan soft film and capsule with the plasticizer mixture of sorbitol and glycerol were studied in this work. The results show that the mixture of plasticizers makes up for the deficiencies of the single plasticizer, improving the quality of the pullulan products. According to the results of TGA, FTIR, XRD, and SEM, it was found that using the plasticizer mixture can improve the thermal stability and compatibility of the pullulan films. The single plasticizer with pure glycerol has a weak connection with the pullulan, improving the fluidity of the pullulan glue, but it reduces the tensile strength of the film. Meanwhile, glycerol has strong hydrophilicity, resulting in poor storage performance of the capsule. The single plasticizer with pure sorbitol improves the crystallinity of the film, resulting in higher tensile strength, but lower elongation at break. Given the excellent elongation at break, temperature stability, and minimal friability exhibited by pullulan products containing a plasticizer mixture of S/G ratio 15:15, along with no significant defects in other characteristics and performance, the soft pullulan capsule with this S/G ratio appears to have strong potential for future applications.

## Figures and Tables

**Table 1:** Effect of S/G ratios on WVP of the films.

S/G	0:30	10:20	15:15	20:10	30:0
WVP/*10^−12^ g·cm·cm^−2^·s^−1^·Pa^−1^	8.95 ± 0.30	6.47 ± 0.15	4.03 ± 0.21	2.68 ± 0.28	1.79 ± 0.17

## Data Availability

The data used to support the findings of this study are available from the corresponding author upon request.
